# How Much Does Stress Cost? A Case–Control Study on Vagally Mediated Heart Rate Variability Responses in Anxious and Non-Anxious Individuals During a Cognitive Task

**DOI:** 10.3390/medsci13030205

**Published:** 2025-09-22

**Authors:** Daniele Chirco, Sara Guidotti, Carlo Pruneti

**Affiliations:** Clinical Psychology, Clinical Psychophysiology, and Clinical Neuropsychology Laboratories, Department of Medicine and Surgery, University of Parma, 43126 Parma, Italy; daniele.chirco@studenti.unipr.it (D.C.); carlo.pruneti@unipr.it (C.P.)

**Keywords:** psychophysical stress, cardiac reactivity, autonomic imbalance, cognition, anxiety

## Abstract

**Background**: Heart rate (HR) and HR variability (HRV) are valid indices of psychophysical stress. Healthy individuals typically exhibit high vagal tone, as indicated by vagally mediated HRV (vmHRV) values. Despite current knowledge, HRV differences between anxious subjects and controls during a cognitive task have not yet been studied. **Methods**: Anxious people were compared to controls through the State–Trait Anxiety Inventory (STAI-Y), both considering State Anxiety (S-Anxiety) and Trait Anxiety (T-Anxiety) one at a time. Subsequently, a psychophysiological stress profile (PSP) was conducted to record HRV values (i.e., SDNN, RMSSD, and HF) at baseline and under induced stress with an electrocardiogram (ECG). During the stress test, the digit span forward task was conducted. **Results**: Significant differences were described by dividing the sample by S-Anxiety in the baseline values of log-HF (t = 2.68; *p* = 0.05; d = 0.85) and log-RMSSD (t = 2.34; *p* = 0.01; d = 0.74). Dividing the sample by T-Anxiety, significant differences were found in the reactivity (t = −2.26; *p* = 0.03; d = −0.70) and recovery (t = 2.11; *p* = 0.04; d = 0.66) log-HF values. Additionally, reactivity log-HF and recovery log-RMSSD values demonstrated significant discriminative power of 68% and 68%, respectively, in accurately identifying individuals with anxiety, as measured by T-Anxiety. Lastly, an association was found between the baseline HR value and the equivalent point of digit span forward in both the anxious (r = 0.59, *p* = 0.01) and control (r = −0.45, *p* = 0.05) groups. **Conclusions**: Although a high vmHRV is considered a protective factor against stress, our findings found that a reduced HRV modulation can distinguish a group of people with significant symptoms of anxiety and hinder cognitive efficiency.

## 1. Introduction

It is well known that heart rate (HR) corresponds to the number of heartbeats per minute, which is variable under the action of different internal and external stimuli, as HR variability (HRV) indicates the variation of heartbeats over time, defined by the interval between two successive heartbeats [1,2]. The analysis of HRV allows the comprehension of the modulating action of the autonomic nervous system (ANS) on cardiac activity [3]. The most precise method to measure HRV is the recording of an electrocardiogram (ECG) [4]. The ECG measures the cardiac electrical activity through electrodes applied to the skin surface. Following the recording, suitable software (e.g., Acqknowledge 3.9, Biopac System Inc., Goleta, CA, USA) extrapolates a tachogram that relates the distance of each single RR interval (the interval between an R wave and the successive wave) as a function of the number of heartbeats [5]. The linear analysis of HRV is mainly based on two methods: time domain analysis and frequency domain analysis [6]. The time domain is calculated based on the normal to normal (NN) interval (i.e., the interval between beats considered normal because they are due to a depolarization of the sino-atrial (SA) node) [7]. Among the parameters most accredited as good indicators of vagal tone are (1) the standard deviation of the NN interval (SDNN); (2) the square root of the mean of the standard deviation of the NN intervals (RMSSD); (3) the NN50 value, which corresponds to the number of NN intervals lasting more than 50 ms; and (4) the pNN50 value, which is the ratio between NN50 and the total number of NN intervals. The most accredited parameters are SDNN and RMSSD. SDNN measures overall HRV over a longer period, reflecting the activity of both the sympathetic (SANS) and parasympathetic branches (PANS) of the ANS, while RMSSD focuses on short-term HRV, mainly related to the activity of the vagus nerve (PANS) [1,7]. The frequency domain analysis is performed using the non-parametric method of the Fourier transform and the calculation of the power spectrum of the tachogram. Values relating to three bands are extrapolated based on the different frequency intervals: (1) very low frequency (VLF), with frequencies between 0.01 Hz and 0.04 Hz, represents the long-term regulation mechanisms, thermoregulation, and hormonal mechanisms; (2) low frequency (LF), with frequencies between 0.04 Hz and 0.15 Hz, represents the sympathovagal oscillations as well as the activity of the baroreceptors; (3) high frequency (HF), with frequencies between 0.15 Hz and 0.4 Hz, is an indicator of the activity of the PANS [8].

Several factors can influence HRV. First, the physiological characteristics of each individual (i.e., sex, age, and circadian rhythm) must be taken into account. Women have a higher vagal tone than men. Furthermore, HRV and vagal tone increase progressively until the age of fifteen, and then, they decrease [8,9]. Concerning circadian rhythm, it has been seen that HRV decreases during the day and increases during the night [10]. Lifestyles and eating habits are parameters to bear in mind as well. For instance, HRV correlates negatively with the body mass index (BMI) but positively with physical exercise [11]. Smoking, alcohol, and coffee significantly impact HRV by increasing adrenergic tone [8], while β-blocking drugs increase parasympathetic tone [12]. In addition to organic pathologies historically characterized by HRV alterations (e.g., myocardial infarction, diabetes mellitus, systemic infections, etc.) [7,8], some psychiatric conditions are associated with reduced HRV (i.e., anxiety and depression) as well [13,14].

HRV is a good indicator of stress levels, as it can quantify the balance between SANS and PANS [15]. Under stress, there is an increase in sympathetic and adrenergic tone, which increases HR and decreases the HF band due to reduced parasympathetic activity [16]. In agreement with Hans Selye’s definition, stress is a “nonspecific response of the body to any demand” and would be part of the so-called general adaptation syndrome. Faced with a stressful stimulus, the body implements a series of neuroendocrine and behavioral adjustments aimed at bringing the organism back to the stage of homeostasis [17].

In the clinical field, anxiety is defined as an emotional state characterized by excessive worry, hypervigilance, and physiological hyperarousal in the absence of an immediate external threat. It may manifest as a response to specific stressors or become pervasive and dysfunctional, as in anxiety disorders, where it significantly interferes with daily functioning [18].

In anxiety, the stress response is frequently activated because the individual’s mental state is persistently marked by apprehension, tension, and discomfort related to the anticipation of potential danger [19]. In particular, within anxious populations, a distinction is made between State Anxiety and Trait Anxiety [19]. The former refers to a transient emotional condition triggered by situational stress, while the latter represents a stable dispositional tendency to experience anxiety across time and contexts.

In both cases, activation of the SANS is reflected in HRV patterns, notably through a decrease in HF components at rest, which are indicative of reduced vagal (parasympathetic) tone [20].

The changes induced by stress are not only autonomic and behavioral but also cognitive. An optimal level of arousal is necessary to obtain good performance, which, if excessive, becomes counterproductive [20]. In this regard, anxiety symptoms determine a reduction in cognitive performance, especially those involving executive functions. As explained by Eysenck and Calvo in 2007, anxiety can interfere with working memory by narrowing and overloading the attentional focus [21]. HRV analysis can be a good indicator of cognitive efficiency [22]. A positive correlation between the value of the HF band at rest and performance has been noted [23]. A 2019 study found that the RMSSD at rest correlates with performance in working memory and attention tasks [24]. At the same time, HR at rest correlates negatively with cognitive functioning [25].

Despite repeated scientific evidence regarding the difference in vagal tone between healthy subjects and subjects with anxiety symptoms, the data are limited when looking only at the baseline values [26,27]. In light of the evidence presented so far, our study aimed to measure the differences in HRV values between subjects with significant symptoms of anxiety and controls, comparing not only baseline values but also reactivity and recovery values induced by stress. Specifically, it was hypothesized that significant differences could exist when considering the values of reactivity and recovery to stress when comparing anxious subjects, similar to the differences found in baseline HRV values. Additionally, the discriminative ability of HRV values in correctly categorizing anxious subjects was analyzed using the ROC curve. Lastly, we also assessed the associations between reactivity and recovery to stress and cognitive performance.

## 2. Materials and Methods

### 2.1. Participants

The study design was observational and case–control. People aged 18 to 65 years were consecutively recruited in our labs. The inclusion criteria in the study were (1) age over 18 years; (2) completion of informed consent; (3) no history of psychiatric and/or neurological syndromes (i.e., previous head trauma, epilepsy, etc.) and/or physical diseases (i.e., sensory disturbances of vision and/or hearing) that could limit the administration of the experimental procedure; no intake of psychotropic drugs with rebound effects on the autonomic nervous system in the last three months (i.e., tricyclic antidepressants, antipsychotics, norepinephrine–dopamine reuptake inhibitors such as bupropion, serotonin modulators such as mirtazapine and trazodone, and serotonin–norepinephrine reuptake inhibitors such as venlafaxine and duloxetine, etc.); (4) no caffeine and no nicotine intake 2 h before and no alcohol assumption 24 h before the registration; and (5) body mass index less than 30 kg/m^2^.

### 2.2. Procedure

Through posters showing an Outlook calendar link, participants were invited to come to the Clinical Psychology, Clinical Psychophysiology, and Clinical Neuropsychology Laboratories of the Department of Medicine and Surgery of the University of Parma. Clinician researchers (with either M.D. and Ph.D.) explained the purpose of the study and the evaluation procedure without specifying the individual phases so as not to influence the experimental method. Subsequently, participants were allowed to book an appointment with a licensed clinical psychologist to receive feedback on the completed questionnaire and psychophysiological reports.

The research followed the 2020 Guidelines for Good Research Practices of the University of Parma. The “REB—Research Ethics Board” considers the Guidelines on Good Practice in Research and the Publication and Dissemination of Results of the University of Parma, issued with DR n. 931 of 3 August 2020. All procedures were conducted in accordance with the 2024 Declaration of Helsinki and the 2005 Universal Declaration on Bioethics and Human Rights regarding research involving human participants. The study was approved by the Ethics Committee of the University of Parma (protocol number: 118310-2024) on 13 May 2024. Informed consent was obtained from all subjects involved in the study.

### 2.3. Measures

Anxiety was measured through the State–Trait Anxiety Inventory—Form Y (STAI-Y) [18], which is a self-report instrument assessing State and Trait Anxiety. The questionnaire consists of 40 items, with answers rated on a four-point Likert scale (1 = not at all, 2 = a little, 3 = sometimes, and 4 = very much). The first 20 items refer to State Anxiety (S-Anxiety), measuring the symptoms experienced by the participant in the actual moment of the administration, while the second 20 items analyze Trait Anxiety (T-Anxiety) by asking how the person feels in general. The score is obtained by summing the scores of the items. Values above 40 points are significantly related to anxiety symptoms. The Italian version of the questionnaire has Cronbach’s alpha indices that fall between 0.91 (for females) and 0.93 (for males) [19].

After ensuring that the subjects enrolled had not consumed coffee, alcohol, or nicotine in the previous two hours, a psychophysiological stress profile (PSP) [28] was recorded. Specific environmental conditions were also monitored (sitting position in a comfortable armchair, feet resting on the floor at 45 degrees, arms resting along the armrests of the chair, and a stable temperature of 19–21 °C). The PSP consists of a 15 min recording of the trend of psychophysiological parameters at rest and under induced stress. To illustrate, the phases include (1) baseline, where the subject is asked to maintain a still position on the chair and close their eyes; (2) the stressor, in which a stressful stimulus is proposed (see below); and (3) rest, where the subject is invited to relax as much as possible. Regarding the psychophysiological parameters, Biopac MP150 (Biopac System Inc., Goleta, CA, USA) was used to collect HR and HRV values. Surface electrocardiogram (ECG) electrodes were placed on the chest at a sampling rate of 1000 samples/s and with limb derivation. ECG was acquired at a rate of 8000 samples/s. Ectopics and artifacts were manually removed from the ECG trace by the control in offline mode. The raw tachogram obtained from the recording was used to analyze HRV through the Kubios HRV scientific analysis software. The HRV indices analyzed in the frequency domain were the low frequency (LF; 0.04–0.15 Hz) and high frequency (HF; 0.15–0.40 Hz) bands obtained by the nonparametric fast Fourier transform (FFT) method. Both measurements contribute to the formulation of the ratio between LF and HF (LF/HF ratio). In addition, time-domain parameters were also collected. Particularly, SDNN and RMSSD were detected. Due to the skewed distribution of LF, HF, and RMSSD values, a natural log transformation was applied (resulting in log-LF, log-HF, and log-RMSSD values, respectively). To obtain the reactivity and recovery values, the recommendations of Laborde et al. [2] were followed. Specifically, reactivity was obtained by calculating the difference between the stress phase and the baseline (i.e., stressor − baseline = reactivity) to quantify changes during the stressor administration (300 s) compared to baseline values (300 s). Similarly, reactivity was obtained by calculating the difference between the rest and stressor phases (i.e., rest − stressor = recovery) to quantify changes during the recovery phase (300 s) compared to the value during stress induction (300 s).

The presentation of the PSP stressful stimulus consisted of the administration of the digit span forward task [29], a neuropsychological test aimed at assessing cognitive performance. This test investigates short-term memory and verbal working memory span, asking the subject to repeat a sequence of digits after the operator [29]. In the first part, the subject is asked to repeat the sequences in the same direction as they were proposed by the operator (digit span forward). In the second part, the subject is asked to repeat the sequence of digits in the opposite direction to the operator (e.g., if the operator proposes “1, 2, 3”, the subject must answer “3, 2, 1”) (digit span backward). The sequences become progressively longer at each series (from three digits up to nine in the forward and up to eight in the backward). The subject can make only one mistake per sequence. The test stops when the subject makes mistakes in both sequences of the series. Scores are assigned based on the last correctly repeated series. Thus, the range of raw scores is from 3 to 9 for the forward and from 3 to 8 for the backward. Raw scores are converted to correct scores, and they are adjusted for age, education, and gender. Further, conversion into equivalent points (EPs) categorizes the correct score into values equal to 0, 1, 2, 3, or 4, where 4 corresponds to the area above the 95% normal threshold and 0 to scores below the 95% tolerance limit. The intermediate values of 1, 2, and 3 correspond to 10.4%, 26.4%, and 50% of the distribution of the normative sample, respectively. Finally, the digit span ratio is the result of the ratio of backward and forward scores. It is assumed that a low ratio score is specifically related to a decrease in executive functions.

### 2.4. Statistical Analysis

SPSS (version 28.0.1.0; IBM Corp, Armonk, NY, USA) was used to perform the planned statistical analyses. The cut-off (40 points) of the STAI-Y was used to allocate participants to the experimental or control group. A comparison of socio-demographic characteristics between groups (i.e., gender, age, marital status, education level, and occupation) was performed using a chi-square test or an independent samples *t*-test.

After ensuring the presence of normal distribution for all variables of interest, independent samples *t*-tests were conducted on the baseline, reactivity, and recovery values of the psychophysiological parameters (i.e., HR, log-HF, and log-RMSSD). Additionally, the discriminative analysis of vmHRV values was ensured. The ability of vmHRV values to discriminate people with higher levels of S-Anxiety and T-Anxiety was assessed through the receiver operator characteristic (ROC) curves. Consistent with the literature [13,14] indicating that reduced vagal activity is associated with elevated anxiety at rest (i.e., baseline and recovery), ROC analyses were performed under the assumption that lower log-HF and log-RMSSD values would predict higher anxiety levels. Conversely, heightened vagal activity under stress (i.e., reactivity) was associated with lower levels of anxiety.

Subsequently, the relationships between the values of the psychophysiological parameters and the scores of the neuropsychological test were identified by calculating the Pearson correlation coefficient. The coefficient of determination between two variables was obtained by the square of their correlation coefficient (r^2^). An r^2^ greater than 0.5 suggests a good or strong association since more than 50% of the variation in one variable can be explained by a variation in the other.

## 3. Results

By considering a standardized Cohen’s effect size of 0.80, a type I error of 5% (α = 0.05), and a type II error of 5% (β = 0.05; power = 95%), an a priori power calculation using GPower 3.1 [30] revealed that 70 participants were required. Considering that the subjects recruited for the research totaled 40, a post hoc power analysis revealed that a sufficient power of 0.80 was achieved, with a large Cohen’s effect size of 0.80.

Table 1 shows the comparison between the anxious (higher scores in S-Anxiety) and the control groups. In this regard, no significant differences emerged in the socio-demographic variables.

Table 2 shows the comparison between the group of anxious people with the controls, divided the sample by T-Anxiety. No significant differences emerged in the socio-demographic variables.

In Table 3, the comparison between the anxious and control groups is shown as divided by S-Anxiety. Significant differences in log-HF (baseline t = 2.68, *p* = 0.05) and log-RMSSD (baseline t = 2.34, *p* = 0.01) were found.

Similarly, Table 4 shows the comparison between the anxious and control groups as divided by T-Anxiety. A significant difference was found in log-HF (reactivity t = −2.26, *p* = 0.03; recovery t = 2.11, *p* = 0.04).

Looking at the sample divided for T-Anxiety scores, the results of the ROC curve analysis were significant for reactivity and recovery values of log-HF and recovery values of log-RMSSD (Table 5).

**Figure 1:**
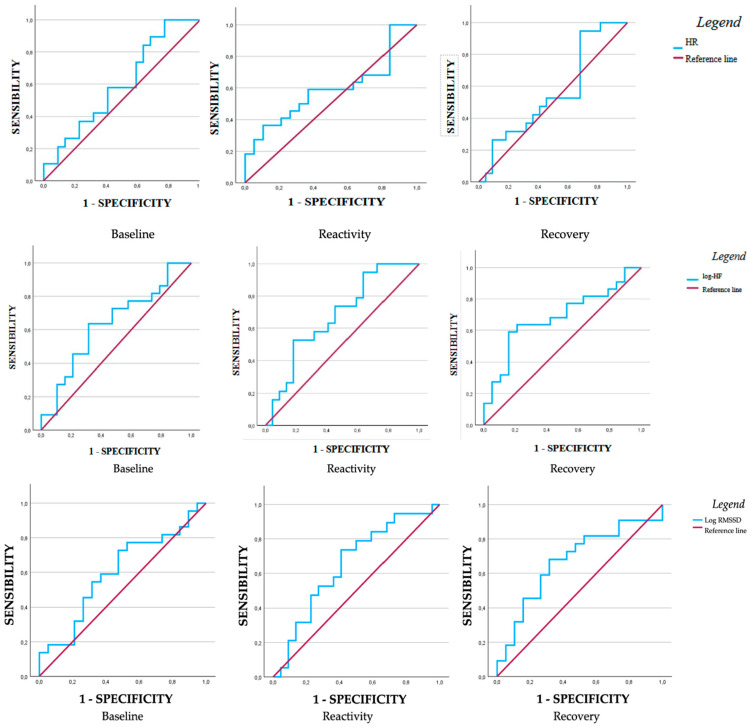
ROC curves of baseline, reactivity, and recovery HR, log-HF, and log-RMSSD values, dividing the sample for T-Anxiety.

In detail, the ROC curve analysis revealed substantial differences in the discriminative ability of the log-HF reactivity and recovery variables. However, only the log-HF reactivity value demonstrated moderate discriminative ability (AUC = 0.68), unlike the recovery value, which had an AUC of 0.32. In contrast, the log-RMSSD recovery value had an AUC of 0.67, which was also statistically significant. Overall, among the three variables examined, log-HF reactivity and log-RMSSD recovery demonstrated positive predictive potential for the outcome of interest, accurately discriminating 68% and 67% of trait-anxious individuals, respectively (Figure 1).

Looking at the sample divided for S-Anxiety scores, the results of the ROC curve analysis were significant for baseline values of both log-HF and log-RMSSD (Table 6).

**Figure 2:**
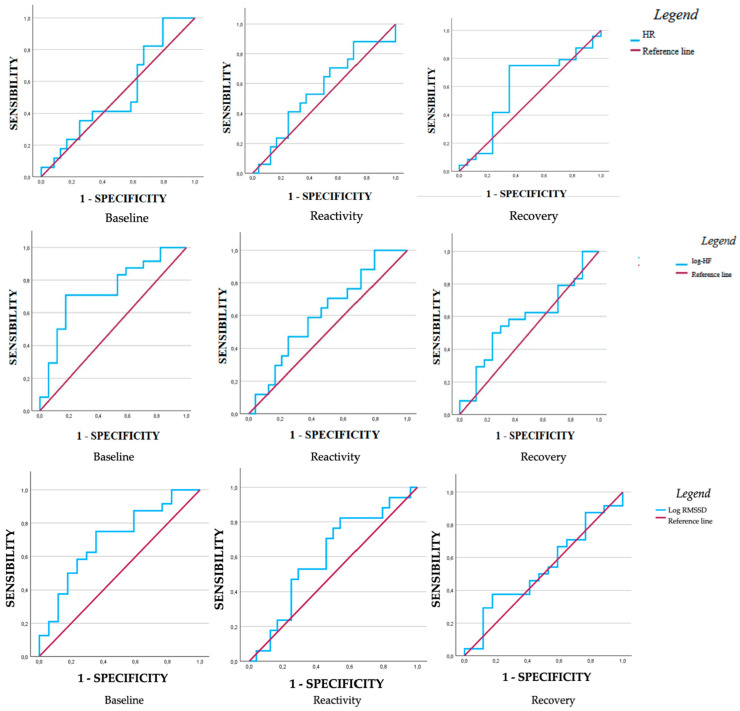
ROC curves of baseline, reactivity, and recovery HR, log-HF, and log-RMSSD values, dividing the sample for S-Anxiety.

Specifically, baseline values for log-HF and log-RMSSD reached statistical significance, demonstrating a reasonable discriminatory ability. The AUC was above 0.70 for both, and they maintained a moderate Youden index (0.53 and 0.40, respectively) (Figure 2).

Lastly, analyzing the correlations between the variables of interest (i.e., HR, HRV, and digit span forward task) within the various groups, only associations between the baseline HR value and the digit span forward task emerged. More specifically, dividing the sample by T-Anxiety, the baseline HR value correlated positively with the equivalent score of the digit span forward in the anxious group (r = 0.59, *p* = 0.01) but negatively in the control group (r = −0.45, *p* = 0.05). Nonetheless, gender and age did not correlate with any of the variables investigated (Significant and non-significant correlations are reported in the Supplementary Table S1).

## 4. Discussion

The study aimed to investigate the association between anxiety symptoms and psychophysiological correlates that support cognitive efficiency. Significant results emerged from the HRV analysis, confirming what is supported by the recent literature that attests to how it is useful to assess the autonomic imbalance associated with the stress response [31]. The subdivision of the sample by S-Anxiety showed significant results regarding the logarithmic values of HF and RMSSD at rest, both reflecting vagal tone. These results are in line with the existing literature on anxiety disorders, as they underline a higher basal activity of the SANS [32].

Equally significant results emerged by dividing the sample by T-Anxiety. A comparison between groups regarding reactivity to stressors administered during PSP revealed greater parasympathetic activity (i.e., log-HF) during stress (i.e., digit span) in the anxiety group. A lower reduction in vagal tone was documented in anxious subjects compared to controls, as if a greater parasympathetic activity was also present under stress that could be interpreted not as a distention but as an exhaustion symptom (e.g., feeling tired, sleepy or lacking energy, along with headaches, dizziness, sore muscles, etc.). The data collected in this work are partially in line with the literature on HRV since, in general, it is suggested that high levels of vagal tone are associated with greater self-regulation, resilience, and adaptability [33]. On the contrary, it is thought that low vagal tone may be indicative of a higher risk of both cardiovascular disease and death [34,35]. Our study obtained results that deviate from this statement when considering trends in HRV values (reactivity and recovery). In our clinical population (i.e., anxious subjects), an increase in vagal tone was noted, especially in the stress phase. HR is thought to increase in response to stress, while parameters representing vagal tone (i.e., HF for the frequency domain and RMSSD for the time domain) are predicted to decrease [16,35,36]. In 2016, Hamilton and colleagues found that increased vagal tone (i.e., HF) was associated with reduced symptoms of depressive exhaustion [37], although it has also been questioned whether this association is recursive [38]. Our results are more in line with those obtained from studies conducted on clinical populations. More specifically, patients with depressive syndromes experienced increased HF in response to stress despite HR reactivity being consistently lower than controls [39]. Furthermore, Liang and colleagues [39] found that the autonomic system shifts toward parasympathetic dominance under stress in depressed subjects. To our knowledge, the unexpected vagal tone pattern has only been observed in the presence of depressive symptoms because studies on other clinical populations are lacking. Our research is the first to describe similar results by dividing a sample based on anxiety symptoms. Nonetheless, log-HF reactivity and log-RMSSD values yielded a discriminative ability of 68% and 67%, respectively, in accurately recognizing people with significant levels of T-Anxiety. Nonetheless, baseline vagal tone (both log-HF and log-RMSSD) demonstrated good accuracy in recognizing people with high levels of S-Anxiety, with good sensitivity values and acceptable Youden indices, confirming previous studies [33].

Finally, interesting results emerged from the correlation analysis between autonomic reactivity and cognitive efficiency, dividing the sample into groups. Although the absence of correlations between the cognitive task and the HRV values does not find consistency in the literature [24,25], a significant association between the values of the HR and the equivalent point of the digit span forward was documented. The higher sympathetic tone associated with a lower cognitive performance in the control group corroborated previous research [26]. However, an inverse trend emerged in the control group. This opposite result could validate the need for anxious subjects to support their cognitive performance with a higher baseline sympathetic arousal, suggesting a probable different psychophysiological functioning in anxious people.

Although the results derived from the present research work are encouraging, our findings must still be read in light of the existing limitations. First, the small sample size suggests the need to interpret the results with caution, and the cross-sectional study design does not allow the definition of the causality of the relationships between the variables of interest. Another key issue is the simplification of HRV, which can lead to a reductionist view [39,40,41].

Despite its limitations, this study raises important questions about the psychophysiological mechanisms involved in anxiety. Autonomic imbalance, particularly reflected in atypical vagal tone modulation, may represent not only a marker of psychological distress but also a potential risk factor for physical conditions, including cardiovascular dysfunctions [42]. In this regard, HF reactivity and recovery values appear more sensitive to T-Anxiety than to S-Anxiety. Specifically, T-Anxiety may become physiologically evident in response to environmental demands that activate the ANS. Thus, HRV analysis could serve as an early, non-invasive indicator of vulnerability to anxiety-related disorders, even before symptoms become evident in resting conditions. From a clinical perspective, HRV and vagal tone modulation in response to stress represent valuable diagnostic indicators, as they reflect an individual’s general capacity to react to environmental challenges and to self-regulate afterwards. This dynamic measurement captures the full engagement of the ANS and may provide deeper insight into the physiological processes underlying acute anxiety episodes. In particular, the abnormal parasympathetic responses observed under stress may mirror the autonomic dysregulation typically seen during panic attacks. Recognizing this pattern early could allow clinicians to detect heightened autonomic sensitivity before the onset of more severe psychopathology, supporting the development of secondary prevention strategies for at-risk individuals. Moreover, interventions such as biofeedback training, which aim to restore a balanced autonomic functioning by enhancing awareness and control over physiological responses, may be particularly effective. By promoting the recalibration of sympathetic and parasympathetic activity, these techniques could help anxious individuals regain autonomic flexibility, improve emotional self-regulation, and prevent stress-related exacerbations. Altogether, these findings reinforce the importance of integrating psychophysiological markers into clinical assessments and early intervention programs, especially for young adults exposed to high levels of stress [43].

## 5. Conclusions

This study explored the associations between cardiac autonomic parameters (i.e., HR and HRV), anxiety dimensions (state and trait), and cognitive performance. The findings revealed that State Anxiety was significantly associated with resting HRV, particularly with vagal tone indices, whereas Trait Anxiety influenced autonomic reactivity and recovery in response to cognitive stress. Although HR did not differ significantly with anxiety levels, it was found to be significantly correlated with performance on the digit span forward task, suggesting a potential link between sympathetic activation and attentional capacity.

These results support the existence of a complex interaction between autonomic functioning, emotional states, and cognitive efficiency, particularly in individuals with elevated anxiety symptoms. From a clinical perspective, the evidence highlights the potential of HRV as a sensitive marker for the early identification of vulnerability to anxiety disorders (especially Trait Anxiety), which may, in turn, have long-term implications for cardiovascular health.

Furthermore, the dual association observed between HR and cognitive performance suggests that anxious individuals may rely on increased autonomic activation to maintain cognitive efficiency, possibly reflecting a compensatory psychophysiological mechanism. Future research with larger and more diverse samples is warranted to confirm these patterns and explore their relevance for personalized interventions and biofeedback-based stress regulation strategies.

## Figures and Tables

**Table 1:** Comparisons of socio-demographic and clinical characteristics between the Anxiety and Control groups divided by S-Anxiety.

Variable		Anxiety Group (*n* = 17)	Control Group (*n* = 24)	*t* or χ^2^	*p*
Age, M (SD)		24.21 (3.69)	25.5 (2.95)	1.23	0.11
Sex, N (%)				0.21	0.65
	Male	9 (22%)	11 (27%)		
	Female	8 (20%)	13 (31%)		
Marital status, N (%)				0.73	0.39
	Unmarried	17 (100%)	23 (96%)		
	Married/cohabitant	0 (0%)	1 (4%)		
Education level, N (%)				1.47	0.69
	High school graduation	7 (17%)	12 (29.3%)		
	University degree	10 (24.4%)	12 (29.3%)		

**Table 2:** Comparisons of socio-demographic and clinical characteristics between the anxious and control groups divided by T-Anxiety.

Variable		Anxiety Group (*n* = 19)	Control Group (*n* = 22)	*t* or χ^2^	*p*
Age, M (SD)		24.10 (3.75)	25.64 (2.77)	1.52	0.06
Sex, N (%)				0.63	0.42
	Male	8 (20%)	12 (29%)		
	Female	11 (27%)	10 (24%)		
Marital status, N (%)				0.88	0.34
	Unmarried	19 (100%)	21 (96%)		
	Married/cohabitant	0 (0%)	1 (4%)		
Education level, N (%)				0.56	0.75
	High school graduation	10 (24.4%)	9 (22%)		
	University degree	9 (22%)	13 (31.6%)		

**Table 3:** Comparisons of reactivity and recovery psychophysiological values between anxious and control groups divided by S-Anxiety.

Variable		Anxiety Group (*n* = 17)		Control Group (*n* = 24)		t (40)	*p*	Cohen’s D
		M	SD	M	SD			
Heart rate (bpm)								
	Baseline	67.55	9.10	65.40	9.44	−0.73	0.23	−0.23
	Reactivity	9.29	6.80	8.63	7.10	−0.30	0.38	−0.09
	Recovery	−9.83	7.06	−8.94	8.30	0.36	0.36	0.11
Log-HF								
	Baseline	6.35	1.07	7.21	0.97	2.68	0.05	0.85
	Reactivity	−0.31	0.55	−0.61	0.80	−1.32	0.10	−0.42
	Recovery	0.24	0.63	0.45	0.74	0.97	0.17	0.30
Log-RMSSD								
	Baseline	3.71	0.54	4.08	0.46	2.34	0.01	0.74
	Reactivity	−0.19	0.30	−0.27	0.35	−0.81	0.21	−0.25
	Recovery	−0.17	0.29	0.22	0.33	−0.43	0.33	−0.14

**Table 4:** Comparisons of reactivity and recovery psychophysiological values between the anxious and control groups divided by T-Anxiety.

Variable		Anxiety Group (*n* = 19)		Control Group (*n* = 22)		t (40)	*p*	Cohen’s D
		M	SD	M	SD			
Heart rate (bpm)								
	Baseline	68.50	9.03	64.38	9.21	−1.44	0.08	−0.45
	Reactivity	6.98	4.53	10.57	8.17	−1.70	0.10	0.53
	Recovery	−7.56	6.44	−10.83	9.37	−1.36	0.18	−0.42
Log-HF								
	Baseline	6.59	1.10	7.08	1.07	1.45	0.15	0.45
	Reactivity	−0.23	0.44	−0.71	0.83	−2.26	0.03	−0.70
	Recovery	0.13	0.49	0.57	0.79	2.11	0.04	0.66
Log-RMSSD								
	Baseline	3.82	0.53	4.02	0.50	1.20	0.24	0.38
	Reactivity	−0.16	0.29	−0.30	0.35	−1.35	0.18	−0.41
	Recovery	0.12	0.26	0.27	0.35	−1.57	0.12	0.49

**Table 5:** ROC curve analysis of HR, log-HF, and log-RMSSD values, dividing the sample for T-Anxiety.

		AUC	SE	CI 95% (LL-UL)	*p*	Sensibility	Specificity	Youden Index
Heart rate (bpm)								
	Baseline	0.60	0.09	0.43–0.77	0.26	0.89	0.78	0.21
	Reactivity	0.59	0.09	0.41–0.76	0.33	0.59	0.36	0.22
	Recovery	0.56	0.09	0.37–0.73	0.53	0.94	0.72	0.22
Log-HF								
	Baseline	0.63	0.09	0.46–0.87	0.80	0.63	0.32	0.32
	Reactivity	0.68	0.08	0.51–0.84	0.03	0.52	0.18	0.34
	Recovery	0.32	0.08	0.15–0.48	0.03	0.59	0.16	0.43
Log-RMSSD								
	Baseline	0.60	0.09	0.43–0.78	0.25	0.72	0.47	0.25
	Reactivity	0.65	0.09	0.48–0.82	0.08	0.73	0.40	0.33
	Recovery	0.67	0.09	0.50–0.84	0.05	0.68	0.32	0.36

Legend: AUC = area under the curve; CI = confidence interval; LL = lower limit; log-HF = high frequencies (logarithmic value); log-RMSSD = root mean square of successive differences (logarithmic value); UL = upper limit; SE = standard error.

**Table 6:** ROC curve analysis of HR, log-HF, and log-RMSSD values, dividing the sample for S-Anxiety.

		AUC	SE	CI 95% (LL-UL)	*p*	Sensibility	Specificity	Youden Index
Heart rate (bpm)								
	Baseline	0.60	0.09	0.34–0.71	0.75	0.82	0.67	0.16
	Reactivity	0.56	0.09	0.37–0.74	0.54	0.88	0.77	0.17
	Recovery	0.59	0.09	0.40–0.77	0.35	0.75	0.35	0.40
Log-HF								
	Baseline	0.73	0.08	0.58–0.89	0.003	0.71	0.18	0.53
	Reactivity	0.61	0.09	0.44–0.79	0.20	0.59	0.37	0.21
	Recovery	0.58	0.09	0.40–0.76	0.36	0.54	0.29	0.25
Log-RMSSD								
	Baseline	0.70	0.08	0.54–0.87	0.01	0.75	0.35	0.40
	Reactivity	0.60	0.09	0.43–0.78	0.25	0.76	0.50	0.26
	Recovery	0.52	0.09	0.35–0.71	0.73	0.37	0.18	0.20

Legend: AUC = area under the curve; CI = confidence interval; LL = lower limit; log-HF = high frequencies (logarithmic value); log-RMSSD = root mean square of successive differences (logarithmic value); UL = upper limit; SE = standard error.

## Data Availability

The data presented in this study are available upon request from the corresponding author, due to the participants’ privacy.

## Supplementary Materials

The following supporting information can be downloaded at: https://www.mdpi.com/article/10.3390/medsci13030205/s1, Table S1: Correlation between Digit span and HRV in clinical group (N = 19).
