# Supplementary material for: How Much Does Stress Cost? A Case–Control Study on Vagally Mediated Heart Rate Variability Responses in Anxious and Non-Anxious Individuals During a Cognitive Task
Source: Med Sci (Basel). 2025 Sep 22;13(3):205. doi: 10.3390/medsci13030205 (PMC12472099; doi:10.3390/medsci13030205)
Supplement: Supplementary file 1 [file medsci-13-00205-s001.zip › medsci-3794131-supplementary.pdf]

Supplementary Table S1 Correlation between Digit span and HRV in clinical group (N = 19).

	Heart Rate			log-HF			log-RMSSD		
	Baseline	Reactivity	Recovery	Baseline	Reactivity	Recovery	Baseline	Reactivity	Recovery
Digit Span Forward									
raw score	0.49*	0.26	-0.29	-0.03	-0.19	-0.17	-0.29	-0.32	-0.03
correct score	0.49*	0.23	-0.26	-0.20	-0.04	0.16	-0.30	0.28	-0.03
equivalent point	0.59**	0.16	-0.28	-0.12	-0.02	0.13	-0.25	0.21	-0.13
Digit Span Backword									
raw score	0.16	0.10	-0.22	0.09	0.10	-0.20	-0.04	0.01	0.09
correct score	0.16	0.07	-0.18	0.07	0.14	-0.21	-0.06	-0.04	0.09
equivalent point	0.24	-0.15	-0.04	-0.16	0.29	-0.29	-0.28	-0.23	0.17
Digit Span Ratio									
raw score	-0.13	-0.01	-0.03	-0.02	-0.02	-0.34	-0.18	-0.11	0.07
correct score	-0.22	-0.10	0.003	0.18	0.18	-0.36	0.12	-0.24	0.13
equivalent point	-0.001	-0.09	-0.04	0.18	0.18	-0.31	0.09	-0.21	0.17

Note: * = $p < 0.05$; ** = $p < 0.01$.
